# Identification of ERβ1 and ERβ2 in human seminoma, in embryonal carcinoma and in their adjacent intratubular germ cell neoplasia

**DOI:** 10.1186/1477-7827-7-56

**Published:** 2009-06-03

**Authors:** Vittoria Rago, Francesco Romeo, Francesca Giordano, Aurora Ferraro, Sebastiano Andò, Amalia Carpino

**Affiliations:** 1 Department of Cell Biology, Faculty of Pharmacy, University of Calabria, Cosenza, Italy; 2 Pathologic Anatomy Unit, Annunziata Hospital, Cosenza, Italy

## Abstract

**Background:**

Estrogens exert a role on germ cell physiology of normal human testis through the mediation of the estrogen receptor (ER) beta subtypes. Epidemiological studies evidenced an increased incidence of testicular germ cell cancer after elevated pre-natal estrogen exposure but the expression of estrogen receptors in these testicular neoplasms has not been well elucidated.

**Methods:**

Immunohistochemistry and Western blot analysis were used to investigate the expression of three distinct ER isoforms, ERalpha, ERbeta1, and ERbeta2 in paraffin-embedded tissues from seminomas and embryonal carcinomas, which are the most common testicular germ cell tumours.

**Results:**

Neoplastic cells of all specimens revealed a positive ERbeta1 and ERbeta2 immunoreactivity, while the ERalpha signal was undetectable. A similar pattern of estrogen receptor immunostaining was also observed in the malignant germ cells of intratubular germ cell neoplasia, adjacent to testicular cancers. Western blot analysis of tumour extracts revealed two immunoreactive bands, a 59 kDa band for ERbeta1 and a 53 kDa band for ERbeta2.

**Conclusion:**

A variable ERbeta expression was previously reported in testicular germ cell tumours and, particularly, an ERbeta down-regulation was evidenced in seminoma and embryonal carcinoma. Conversely, the current study has clearly identified ERbeta1 and ERbeta2 in the neoplastic cells of seminoma and embryonal carcinoma, as well as in the malignant cells of their common pre-invasive precursor, intratubular germ cell neoplasia. Therefore, our findings suggest that ERbeta1, together with a possible ERbeta2 contribute, can mediate estrogen action in both early and late neoplastic testicular germ cells, not confirming the previously hypothesized antiproliferative effect of ERbeta on male gonadal cells.

## Background

A current worldwide rise in the incidence of testicular germ cell cancer has been evidenced by epidemiological studies [1]. However, despite of growing researches on pathogenesis of these testicular neoplasms, lots of gaps of knowledge remain on molecular mechanisms responsible for the tumorigenesis process [2].

An increased rate of testicular germ cell tumours, after prenatal estrogen exposure, has supported the hypothesis that these hormones could play a role in the male gonadal carcinogenesis in combination with genetic susceptibility [3-6]. On the other hand, the recognized ability of estrogens to regulate cell growth and apoptosis [7,8] could be involved in testicular neoplastic proliferation.

Estrogens exert their pleiotropic and tissue-specific effects through the differential expression of distinct estrogen receptors, ERα and ERβ, and their coregulators [9].

In normal human testis, estrogen receptor-β is known as the mediator of physiological estrogen action. Testicular somatic and germ cells express mainly the two isoforms ERβ1 and ERβ2 [10-12]. ERβ1 is the full length wild type ERβ and the only fully functional isoform [13] while ERβ2 is a splice variant, lacking aminoacid essential for binding estradiol [14]. Mediation of estrogen signalling is determined by differential dimerization of ERs and a recent study indicated that ERβ2 can heterodimerize with ERβ1 and enhance its transactivation in a ligand-dependent manner [13]. However, the distinct biological roles of the ERβ isoforms is still undefined [15].

In testicular germ cell tumours the expression of estrogen receptors has not been still clarified. In fact, a highly variable ERβ expression has been reported in the different tumour types [16].

Therefore, with the aim to expand the ER knowledge in male gonadal neoplasms, the present work investigated the expression of three distinct ER isoforms (ERα, ERβ_1 _and ERβ_2_) in testicular tissues from patients with seminoma and embryonal carcinoma, which are the most common cancers in young men.

## Methods

### Patients

The investigation has been performed on formalin-fixed and paraffin-embedded testis tissues from 16 male patients (ages 20 – 35 years) with pure seminoma (n = 10) and pure embryonal carcinoma (n = 5) undergoing to therapeutic orchidectomy. Control testicular tissues were obtained from 2 male patients (ages 29 and 35 years) showing testes with a like-Sarcoidosis granulomatous lesion. The archival cases were provided by the Pathologic Anatomy Unit (Annunziata Hospital, Cosenza, Italy). The ethical committee members of the University of Calabria approved the investigation programme.

### Histopathological analysis

Morphological studies were carried out by Haematoxylin-Eosin staining. Testis tumour samples were also investigated by placental-like alkaline phosphatase (PLAP) immunohistochemistry, using the anti- human PLAP as primary antibody (30 minutes at RT) followed by the avidin-biotin-horseradish peroxidase complex (ABC/HRP) and diaminobenzidine visualization.

#### Chemicals

The reagents were purchased from Sigma Aldrich (Milan, Italy), unless otherwise indicated.

### Antibodies

Anti-ERα primary antibody was mouse monoclonal F-10 (Santa Cruz Biotechnology, California, USA) which recognizes epitope mapping at the C-terminus region of the human native ERα. Anti-ERβ primary antibody was rabbit polyclonal H150 (Santa Cruz Biotechnology, California, USA) which recognizes epitope mapping at the N-terminus regions of human native ERβ. Anti-ERβ1 primary antibody was mouse monoclonal MCA1974S (Serotec, Oxford, UK) which recognizes epitope mapping at the C-terminus regions of human native ERβ1 while anti-ERβ2 primary antibody was mouse monoclonal MCA2279S (Serotec, Oxford, UK) which recognizes epitope mapping at the C-terminus regions of human native ERβ2. Anti human PLAP antibody was monoclonal mouse, clone 8A9, (DAKO-Cytomation, Milan, Italy). Rabbit polyclonal anti β-actin (Santa Cruz Biotechnology, California, USA) was also used as loading control. Biotinylated goat-anti-mouse IgG (Vector Laboratories, INC, Burlingame, California, USA), goat anti-rabbit horseradish peroxidase conjugated IgG (Amersham, USA) and goat anti-mouse horseradish peroxidase conjugated IgG (Amersham, USA) were used as secondary antibodies.

### Immunohistochemical analysis

Paraffin embedded sections, 5 μm thick, were mounted on slides precoated with poly-lysine, and then they were deparafinized and dehydrated (7–8 serial sections). Immunohistochemical experiments were performed after heat-mediated antigen retrieval in 0.01 M citrate buffer, pH 6. Hydrogen peroxide (3% in distillate water) was used, for 30 minutes, to inhibit endogenous peroxidase activity while normal goat serum (10%) was utilised, for 30 minutes, to block the non-specific binding sites.

Immunodetection was carried out using anti-ERα (1:50), anti-ERβ1 and anti-ERβ2 (1:100) primary antibodies at 4°C overnight. Then, a biotinylated goat-anti-mouse IgG was applied (1:600) for 1 hour at RT, followed by the avidin-biotin-horseradish peroxidase complex (ABC/HRP) (Vector, Laboratories, CA, USA). Immunoreactivity was visualized by using the diaminobenzidine chromogen (DAB)(Zymed Laboratories, CA, USA). Testis sections were also counterstained with haematoxylin. The primary antibody was replaced by normal rabbit serum in negative control sections. Absorption controls have utilised primary antibodies preabsorbed with an excess (5 nmol/ml) of the purified respective blocking peptides (Santa Cruz Biotechnology, Ca, USA), at 4°C for 48 hours. Breast cancer tissue was used as positive control for ERα, while control human testis was used as positive control for both ERβ1 and ERβ2. Six-seven serial sections were processed for each sample.

### Scoring system

The immunostained slides were examined by light microscopy using the "Allred Score" [17], which combines a proportion score and an intensity score. A proportion score was assigned representing the estimated proportion of positively stained tumour cells (0 = none; 1 = <1/100; 2 = 1/100 to <1/10; 3 = 1/10 to <1/3; 4 = 1/3 to 2/3; 5 = >2/3). An intensity score was assigned by the average estimated intensity of staining in positive cells (0 = none; 1 = weak; 2 = moderate; 3 = strong). Proportion score and intensity score were added to obtained a total score that ranged from 0 to 8. A minimum of 100 cells were evaluated in each slide. Six-seven serial sections were scored for each sample. The one-way ANOVA was used to evaluate the differences in the scores between tumoral and control samples.

### Protein extraction

Protein extraction from formalin-fixed paraffin-embedded sections has been carried out according to Ikeda [18]. Briefly, 50 μm testis sections were deparaffinized in xylene, dehydrated in graded ethanol, immersed in distilled water, and air dried. Then, the neoplastic area was recovered from the glass slides, further it was cut into small pieces and placed in Eppendorf tubes. Two hundred μl of RIPA buffer, pH 7,6 (1 M NaH_2_PO_4_, 10 mM Na_2_HPO_4_, 154 mM NaCl, 1% Triton X-100, 12 mM C_24_H_39_O_4_Na, 0,2% NaN_3_, 0,95 mM NaF, 2 mM PMSF, 50 mg/ml aprotinin, 50 mM leupeptin) containing 0,2% SDS, was added to each tube and the contents were incubated at 100°C for 20 minutes, followed by incubation at 60°C for 2 hours. After incubation, tissue lysates were centrifuged at 15,000 × g for 20 minutes at 4°C and the supernatants were stored at -80°C until biochemical analysis.

### Western blot analysis

Tissue lysates were quantified using Bradford protein assay reagent [19]. Equal amounts of protein (50 μg) were boiled for 5 minutes, separated under denaturing conditions by SDS-PAGE on 10% polyacrylamide Tris-glycine gels and electroblotted to nitrocellulose membrane. Non-specific sites were blocked with 5% non fat dry milk in 0.2% Tween-20 in Tris-buffered saline (TBS-T) for 1 hour at room temperature and incubated overnight with anti-ERα(F-10) (1:500), anti-ERβ(H150) (1:500), anti-actin (1:1000) primary antibodies. The antigen-antibody complexes were then detected by incubation of the membranes for 1 hour at 22°C with the horseradish peroxidase-conjugated secondary antibodies (1:7000). The bound secondary antibodies were located with the ECL Plus Western blotting detection system (Amersham, USA) according to the manufacturer's instruction. Each membrane was exposed to the film for 2 minutes.

Protein extracts from cultured MCF7 (breast cancer cell line) and LnCap (prostate cancer cell line) cells were used as positive controls for ERα and ERβ respectively. Cells were maintained at 37°C in a humidified atmosphere of 5% CO2 in air and were cultured in DMEM-F12 (MCF-7 cells) or in RPMI-1640 medium (LnCap cells) supplemented with L-glutamine (2 mM), penicillin (100 U/ml), streptomycin (100 U/ml), and 10% fetal bovine serum (Life Technology, Milan, Italy)

Negative controls were prepared using tissue lysates, where antigens were previously removed by pre-incubation with specific antibodies (1 hour at room temperature) and subsequently immunoprecipitated with protein A/G -agarose.

## Results

### Histopathological study

#### Seminoma

**Figure 1:**
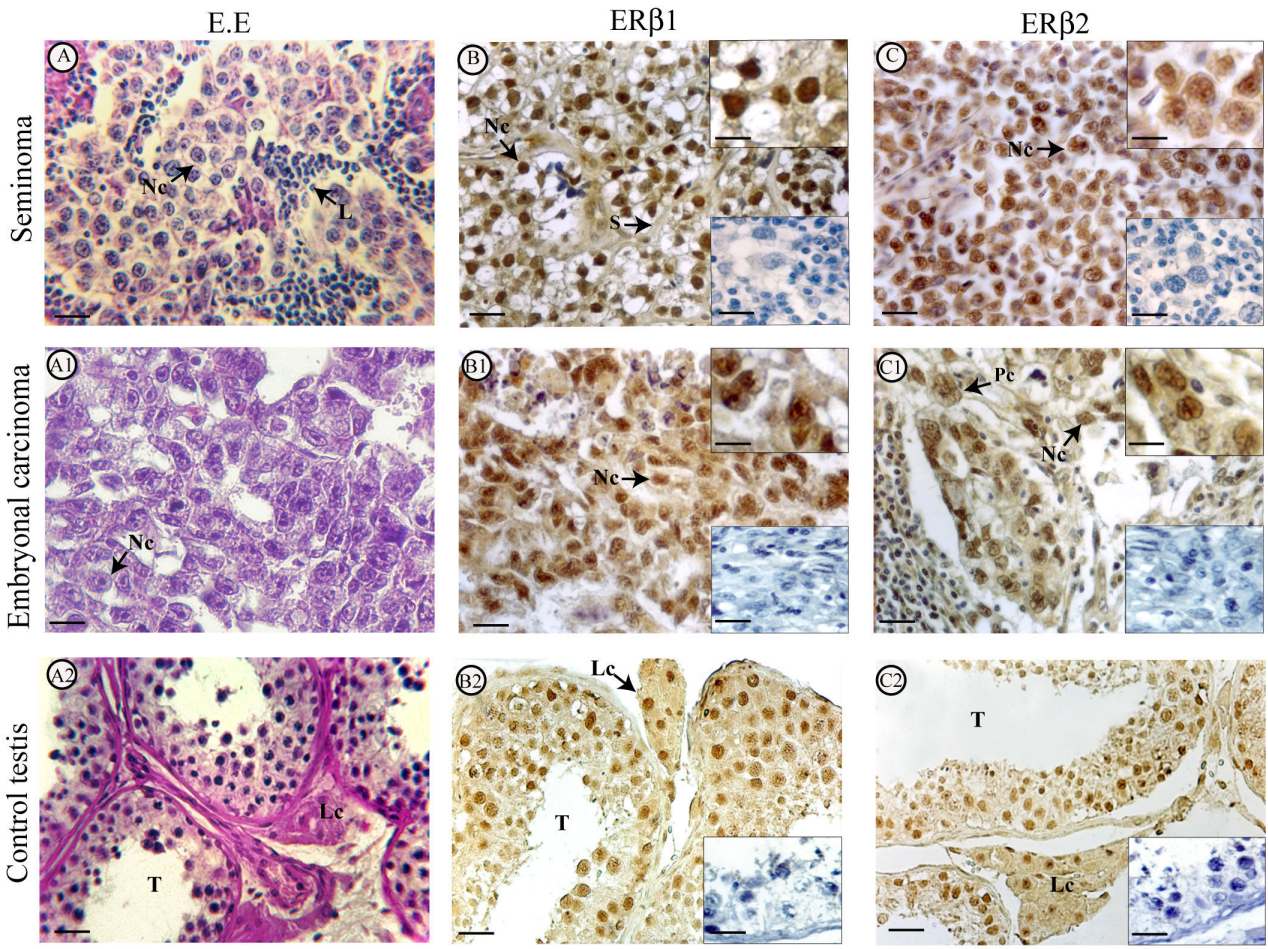
**Morphology and immunolocalization of estrogen receptors (ERβ1, ERβ2) in seminoma, embryonal carcinoma and control testis**. The pictures show representative samples. A, A1, A2: Haematoxylin-Eosin staining. B, B1, B2: ERβ1 immunolocalization. C, C1, C2: ERβ2 immunodetection. Scale bars = 12,5 m μ. L, linfocytes. Lc, Leydig cell. Nc, neoplastic cell. Pc, plurinuclear cell. S, septa. T, seminiferous tubule. Inserts at the bottom right side (B, B1, B2, C, C1, C2): absorption controls. Scale bars = 12,5 mμ. Inserts at the top right side (B, B1, C, C1): higher magnification of the pictures. Scale bars = 5 mμ.

Morphological analysis of pure seminoma samples (stages I) showed the presence of typical tumoral tissues with uniform populations of large neoplastic cells which were arranged in diffuse sheets separated by thin septae. Extensive leukocyte infiltrations were observed in all samples (Fig 1A shows a representative sample).

#### Embryonal carcinoma

Tumoral tissues of pure embryonal carcinoma showed nodular areas surrounded by connective tissue. The large neoplastic cells had ill-defined borders, big nuclei, pale cytoplasms and were mainly arranged in glandular pattern. Plurinuclear cells were also present (Fig 1 A1 shows a representative sample).

#### IGCN

**Figure 2:**
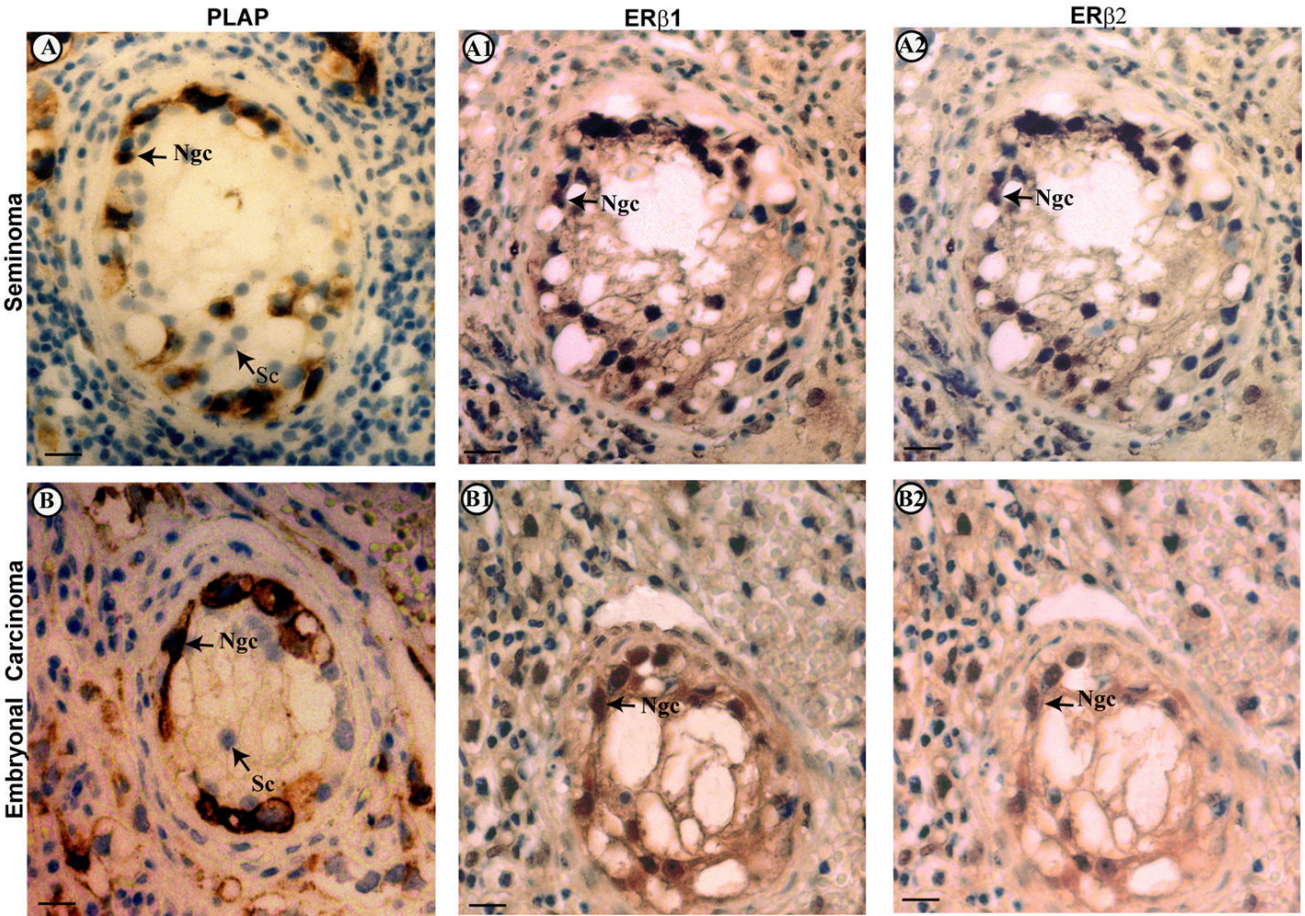
**PLAP staining and immunolocalization of estrogen receptors (ERβ1, ERβ2) in intratubular germ cell neoplasia adjacent to seminoma (A) and embryonal carcinoma(B); the pictures show representative samples**. A, B: PLAP staining. A1, B1: ERβ1 immunolocalization. A2, B2: ERβ2 immunodetection. Ncg, neoplastic germ cell. Sc, Sertoli cell. Scale bars = 5 mμ.

The regions adjacent to seminoma (70% of samples) and to embryonal carcinoma (60% of samples) have shown the presence of seminiferous tubules lacking spermatogenesis, which were identified as intratubular germ cell neoplasia (IGCN), unclassified type. These intratubular lesions were characterized by the basal proliferation of undifferentiated atypical enlarged germ cells, with big nuclei, and by the presence of Sertoli cells in luminal displacement. Cytoplasmic and membranous dark staining have revealed the PLAP immunopositivity in the malignant cells aligned along the basal portion of seminiferous tubules (Fig 2A, B), indicating their germ cell origin.

### Immunohistochemistry

Immunostainings of all seminoma and embryonal carcinoma specimens revealed an intense nuclear ERβ1 and ERβ2 immunoreactivity of neoplastic cells while the leukocytes were immunonegative (Fig 1:B, C, B1, C1)

**Figure 3:**
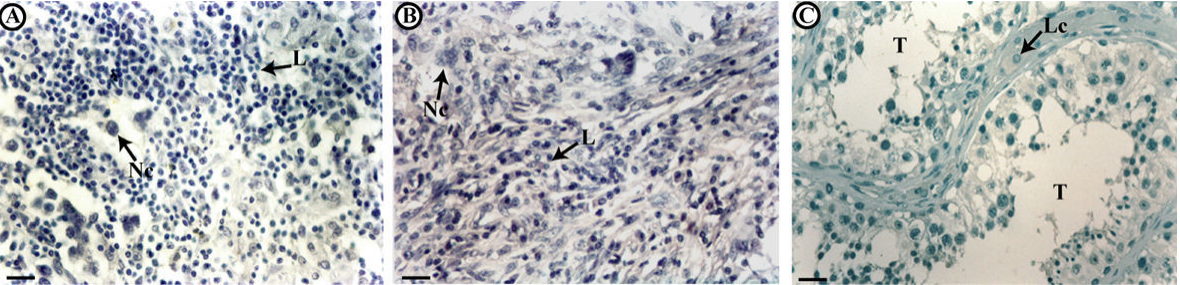
**Immunolocalization of ERα in seminoma (A), embryonal carcinoma(B) and control testis (C); the pictures show representative samples**. L, linfocytes. Lc, Leydig cells. Nc, neoplastic cell. T, seminiferous tubules. Scale bars = 12,5 mμ.

As expected, in control testis sections the cells of tubular and interstitial compartments showed strong exclusively nuclear ERβ1 and ERβ2 immunostainings (Fig 1:B2, C2). ERα signal was totally absent in control and tumoral testes) (Fig 3A, B, C).

Table 1 shows the intensity staining scores of tumoral and control samples. Statistical analysis evidenced that ERβ1 score of seminomas was higher than ERβ1 score of control testes (p < 0.05).

**Table 1:** Immunostaining scores (Allred score) of ERβ1 and ERβ2 in tumoral and control testes

**Groups**	**ERbeta1**	**ERbeta2**
*seminoma*		
Patient 1	8	7
" 2	6	5
" 3	7	6
" 4	7	6
" 5	7	6
" 6	8	6
" 7	7	7
" 8	8	5
" 9	7	6
" 10	8	6
*embryonal carcinoma*		
Patient 1	6	5
" 2	6	5
" 3	7	6
" 4	7	5
" 5	7	6
*control testis*		
Patient 1	6	5
" 2	6	5

Immunostained slides scores as follows: Total score= Proportion score+ Intesity score (range 0–8)

In addition, neoplastic cells of intratubular germ cell neoplasia (IGCN), adjacent to seminoma and embryonal carcinoma, revealed a positive ERβ1 and ERβ2 immunostaining (Fig 2: A1, A2, B1, B2) but no ERα immunoreactivity (data not shown).

All tissues used as positive controls showed a strong ERα/ERβ1/ERβ2 immunoreactivity (data not shown). Furthermore, negative controls (data not shown) and absorption controls (inserts) were all immunonegative.

### Western blot analysis

**Figure 4:**
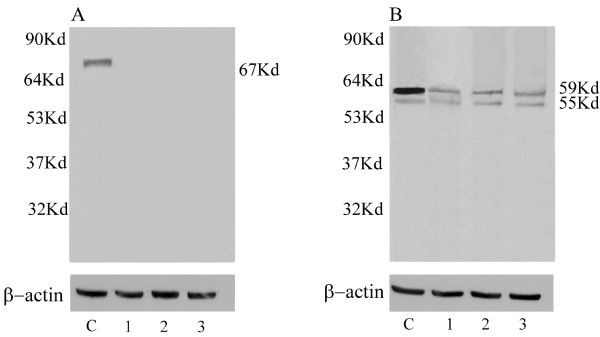
**Immunoblots of estrogen receptors (ERα, ERβ1, ERβ2) from representative protein extracts of seminoma and embryonal carcinoma**. ERα immunoblot (A): MCF7 cultured cells *(C)*, seminoma *(lane 1*), embryonal carcinoma *(lane 2) *and control testis *(lane 3*). ERβ1- ERβ2 immunoblot (B): LnCap cultured cells *(C)*, seminoma *(lane 1*), embryonal carcinoma *(lane2) *and control testis *(lane 3*). β-actin serves as a loading control. *Numbers *on the left-hand side correspond to the standard molecular weights *Numbers *on the right-hand side correspond to molecular weights of the detected protein.

For WB analysis of tumour extracts we have used a single ERβ antibody which was tested for this application. This anti-ERβ antibody, mapping at the N-terminus region of human native ERβ, let us to reveal both the ERβ subtypes. In fact, the immunoblots of seminoma and embryonal carcinoma extracts showed a thick ERβ1 band at ~59 kDa and a thin ERβ2 band ~53 kDa (Fig 4B: *lanes 1, 2*). Similar bands were observed in the LnCap cell extract, used as positive control (Fig 4B: *lane C*). Conversely, using an antibody against ERα, no protein band has been observed in the immunoblots of tumoral testes (Fig 4A: *lanes 1, 2*), while MCF7 cell extract (positive control) showed the classical ~67 kDa band (Fig 4A: *lane C*). As expected, control testes showed the ~59 kDa ERβ1 band and the ~53 kDa ERβ2 band (Fig 4B: *lane 3*) but not the ERα band (Fig 4A: *lane 3*)All the negative control lanes were unlabelled (data not shown).

## Discussion

Testicular germ cell tumours are joined to cryptorchidism, hypospadias and impaired spermatogenesis in the "testicular dysgenesis syndrome" (TDS), which includes disorders resulting from the impairment of fetal gonadal development [20]. In normal human testis, fetal gonocytes change into neonatal spermatogonia which mature into viable sperm after puberty. Conversely, testicular germ cell tumours appear to arise from abnormal gonocytes which arrest their differentiation, undergoing a malignant transformation [21,22].

Increased estrogen exposure could be an important aetiological factor to promote the TDS disorders and particularly testis cancer [6], but the related molecular mechanisms remain to be elucidated.

Estrogen receptors orchestrate both transcriptional and non genomic cellular responses to estrogens. Therefore, they are involved in physiological germ cell differentiation, but, at the same time, they could mediate the estrogen signalling on malignant germ cell proliferation, probably damaging DNA such as reported in breast cancer [23].

Estrogen receptor-β mediates physiological estrogen action in somatic and germ cells of normal human testis [10-12], but its possible involvement in estrogen-induced carcinogenicity has been also reported. In fact, experimental estrogen exposure of rat testis resulted in oxidative DNA damage via an ERβ-mediated process [24].

The present work has identified ERβ1 and ERβ2 in neoplastic cell nuclei of seminoma and embryonal carcinoma, which are the most common testicular germ cell tumours. Agreeing with Saunders's report [11], a nuclear staining of the two ERβ isoforms has been also observed in control germ cells. Western blot analysis of tumour extracts confirmed these findings showing two protein bands consistent with the ERβ1 and ERβ2 molecular weights.

Two previous studies detected differentially ERβ1 in testicular germ cell tumours. In fact, ERβ expression was strong in endoderma sinus tumour and in teratoma [16] but it was low in seminoma and in embryonal carcinoma [16,25]. Furthermore, a punctuate cytoplasmic ERβ localization has been reported in three seminoma tumoral fragments as well as in seminoma cell line (JKT-1) [26]. Therefore, previous data are conflictual with respect to our results which have evidenced a remarkable and undoubted ERβ1 and ERβ2 immunoreactivity in the nuclei of seminoma and embryonal carcinoma cells. These discrepancies could be due to the variability in protein expression of different tumour samples and/or to the use of different antibodies. Despite of previous studies, the present investigation has performed immunohistochemical experiments using primary antibodies which were specific for each of the two ERβ isoforms and tested for imminohistochemistry.

Our data do not support a possible role of the ERβ down regulation in testicular tumorigenesis as hypothesized by the previous authors; however, as these results are conflictual, further studies need to confirm them.

In addition, our work has shown the ERβ1 and ERβ2 expression in the big neoplastic cells (recognized by PLAP) of IGCN which are considered the pre-invasive precursors of testicular germ cell tumours. In fact, phenotypic similarity of IGCN cells with fetal gonocytes, embryonic stem cells and primordial germ cells [20,27-29] suggested that IGCN cells are originated from the arrest of fetal germ cell differentiation followed by malignant transformation. Therefore, our paper has revealed, for the first time, the expression of ERβ1 and ERβ2 in the early stages of testicular tumorigenesis process. Interestingly, the two ERβ isoforms were identified in human fetal gonocytes [30], but with a clear prevalence of ERβ2 on ERβ1.

Previous studies from our laboratory demonstrated aromatase expression in tumoral cells of seminoma and adjacent IGCN [31], as well as in embryonal carcinoma [unpublished data], indicating a local estrogen production. In the current study, seminoma and embryonal carcinoma cells, expressing ERβ1 and ERβ2 but not ERα, have shown an ER pattern similar to that one of normal testicular cells. A high ERβ1 immunoreactivity of seminoma cells has been detected, but this new finding needs to be confirmed in a larger size of sample set, before speculating its involvement in the autocrine/paracrine estrogen action promoting testicular germ cell malignancies.

Conversely, our recent paper evidenced an altered ER expression pattern in two human Leydig cell tumours, suggesting the possible contribute of the unexpected ERα presence to the tumour cell growth and progression [32]. A recent work [33] suggested that estrogens could contribute to human testicular germ cell cancer proliferation by a rapid activation of ERK1/2 and PKA through a non classical membrane ER. Therefore, further studies will be necessary to expand the knowledge of estrogen signalling involved in testicular tumorigenesis.

## Conclusion

The present study has demonstrated the expression of ERβ1 and ERβ2 in seminoma and embryonal carcinoma cells, as well as in neoplastic cells of their pre-invasive precursor, IGCN. Therefore, our findings suggest that the two ERβ subtypes can mediate estrogen action in both early and late neoplastic testicular germ cells, not confirming a possible suppressive role of ERβ on male gonadal cell proliferation, as previously hypothesized.

## Competing interests

This work was supported by MURST and Ex 60% 2008, and PRIN 2008.

## Authors' contributions

VR: the author responsible for performing the immunohistochemical expriments and participating in the analysis and interpretation of data.

FR: the author responsible for histoplathological diagnosis.

FG: the author responsible for performing Western blot analysis.

AF: the author responsible for sample collection.

SA: the author responsible for a critical revision of the manuscript.

AC: the author responsible for conception, design, analysis and interpretation of data as well as of drafting manuscript.

All authors read and approved the final manuscript
